# Genetic uniformity and occurrence of *Bartonella bovis* in European cattle: Indications of exophagic transmission

**DOI:** 10.1016/j.parepi.2026.e00532

**Published:** 2026-08-14

**Authors:** Iva Hammerbauerová, Kristýna Dulavová, Petr Václavek, Jan Votýpka

**Affiliations:** a Department of Parasitology, Faculty of Science, Charles University, Prague, Czechia; b Department of Virology, State Veterinary Institute, Jihlava, Czechia; c Institute of Parasitology, Biology Centre, Czech Academy of Sciences, České Budějovice, Czechia

**Keywords:** Vectors, Zoonoses, Bartonella, Ectoparasites, Blood parasites

## Abstract

*Bartonella bovis* is an intracellular bacterium infecting cattle, with competent vectors yet to be determined. We compared *B. bovis* prevalence between pasture-raised and indoor-housed cattle to assess potential vector candidates. A total of 548 blood samples from Czech cattle and 48 samples from cattle from southeastern Romania were analyzed using qPCR (*ssrA* gene) and nested PCR (*gltA* gene). In pasture herds, 18.39% (64/348) of samples were positive, all but one representing a single *B. bovis* genotype identical to isolates from Brazil, Guatemala, and France. In Romanian herds, also reared outdoors, prevalence was 12.5% (6/48) mostly matching Czech genotypes. These results indicate low genetic diversity of *B. bovis* compared to other *Bartonella* species (e.g., *B. schoenbuchensis* in red deer), supporting a clonal population structure. In contrast, indoor cattle were completely negative (0/200). The exclusive occurrence of *B. bovis* in pasture-raised cattle and their exposure to blood-feeding arthropods suggests transmission by exophagic vectors, possibly horn flies (*Haematobia irritans*) or horseflies (Tabanidae). Overall, our findings, while not demonstrating the competence of a specific vector, provide epidemiological indications that outdoor exposure supports vector-borne transmission and narrows the range of plausible candidate vectors in Europe.

## 1 Introduction

*Bartonella* species are a group of facultative intracellular bacteria transmitted by blood-feeding arthropods, which infect red blood cells and endothelial cells of a wide range of mammals, including humans and domestic animals, in whom they can cause both mild and severe pathologies. Their role as potential agents of zoonotic diseases has been intensively studied only in recent years; however, many uncertainties remain regarding their occurrence, transmission, and effects in the host. In cattle, the most frequently detected species is *Bartonella bovis*, whose prevalence varies considerably between regions – from 15.3 to 20% in North and West Africa (Boularias et al., 2020; Raoult et al., 2005) to 70.4–82.4% in North and South America (Cherry et al., 2009; Saisongkorh et al., 2009). The animals' age matters as well; calves seem to be *Bartonella*-free, while heifers have the highest prevalence (Maillard et al., 2006). While most *Bartonella* infections in animals are thought to be asymptomatic, *B. bovis* has been linked to several cases of endocarditis in cows (Erol et al., 2013; Maillard et al., 2007), as well as a clinical case in a veterinarian (Gamboa-Prieto et al., 2023). Other species occasionally found in cattle include *B. schoenbuchensis* subsp. *chomelii* (Maillard et al., 2004) and, more rarely, *B. schoenbuchensis* subsp. *schoenbuchensis* (Bai et al., 2013).

Epidemiologically, competent vectors are crucial for the pathogen's life cycle, yet for *B. bovis*, despite its ubiquity in cattle and possible veterinary significance, they remain unknown. It has not been detected in keds (Hippoboscidae), which transmit the related *B. schoenbuchensis* (Dehio et al., 2004; Sato et al., 2021). Among the varied cattle ectoparasites tested – horn flies, stable flies, deer flies, horse flies, ticks, and cattle tail lice – *B. bovis* DNA has only been detected in horn flies and ticks, with very low prevalences (Boularias et al., 2020; Chung et al., 2004; Gutiérrez et al., 2014; Kho et al., 2015; Seo et al., 2025; Tsai et al., 2011). However, the mere detection of pathogen DNA in a blood-feeding arthropod is insufficient and can be misleading due to ingested host blood. While the identification of a competent vector is methodologically challenging and usually requires experimental verification, epidemiological studies comparing host groups exposed to different potential vectors can provide valuable insights. In Czechia, intensive dairy cattle farming is typically based on large herds, with lactating cows usually housed indoors throughout the year. In some farms, only selected categories, particularly heifers and/or dry cows, may have temporary access to outdoor enclosures, while biosecurity measures, including controlled animal introductions, restricted farm access, separation of animal groups and routine sanitation, are applied similarly to other intensive dairy systems. The presented study compared *Bartonella* occurrence in cattle reared on outdoor pastures with cattle kept entirely indoors for insights on vector ecology.

## 2 Materials and methods

**Fig. 1:**
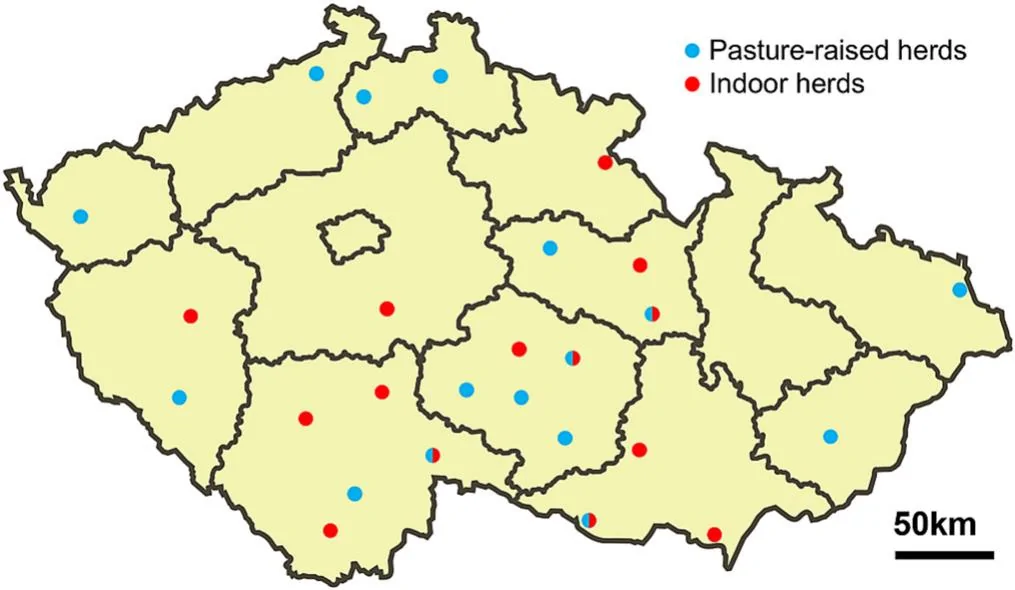
Map of the sampled locations for both pasture-raised and indoor herds.

Across various biotopes and regions in Czechia, 38 cattle farms were selected for sampling. A total of 18 herds consisted of cattle reared on open pastures, while the remaining herds were kept entirely indoors. Fig. 1 shows a map of the sampled locations. From each herd, blood samples were collected from randomly selected adult cows; 20 cows from pasture herds, 10 cows from indoor herds. However, for two herds grazing outdoors, only 14 animals could be sampled due to technical constraints. Sampling was conducted in September–November. Despite our efforts to ensure balanced representation and comparability between pasture-raised and indoor-housed cattle, this study could not account for other potential factors, such as herd management (e.g., replacement practices and animal origin), breed, insecticide or ectoparasitic treatments, and regional climate or habitat. As an external comparison to the Czech herds, 48 cattle from two free-ranging herds in southeastern Romania were included.

DNA was extracted using the commercial High Pure PCR Template Preparation Kit (Roche, Basel, Switzerland) following the protocol for isolation of nucleic acids from mammalian tissue. For qPCR, a set of primers (ssrA-F, 5′-GCTATGGTAATAAATGGACAATGAAATAA-3′; ssrA-R, 5′-GCTTCTGTTGCCAGGTG-3′) and a probe (6FAM-5′-ACCCCGCTTAAACCTGCGACG-3′-BHQ1) targeting the *ssrA* gene (transfer mRNA) was used (Diaz et al., 2012), each at a concentration of 10 μM. qPCR reactions were prepared in a sterile laminar-flow cabinet using 96-well PCR plates. The reaction mix and PCR-grade water were sourced from the LightCycler® 480 Probes Master kit (Roche, Basel, Switzerland). Real-time PCR was performed on the LightCycler® 480 Real-Time PCR System (Roche, Basel, Switzerland) instrument operating with LightCycler® 480 software version 1.5.1.62, with the following thermocycling parameters: one pre-incubation cycle of 95 °C for 5 min followed by 50 amplification cycles of 95 °C for 10 s and 60 °C for 35 s, terminating with one cooling cycle of 40 °C for 10 s.

A random subset of samples (*n* = 116) was additionally tested using semi-nested PCR targeting the commonly used genotyping gene *gltA*, using the primers gltA443-F-D (5’-GCYATGTCTGCATTYTATCA-3′), gltA1210-R (5’-GATCYTCAATCATTTCTTTCCA-3′) and gltA1137n (5’-AATGCAAAAAGAACAGTAAACA-3′), PCR mix concentrations and thermocycling protocols according to (Hammerbauerová et al., 2025). PCR products were purified using a commercial kit containing two enzymes – FastAP Thermosensitive Alkaline Phosphatase and Exonuclease I (both Thermo Scientific). Sequenced samples were analyzed using Geneious Prime 2025.2.2 (https://www.geneious.com).

## 3 Results

A total of 348 blood samples from 18 herds of pasture-raised cattle and 200 samples from 20 indoor herds were analyzed. All samples were tested using qPCR targeting the *ssrA* gene, and approximately half of the randomly selected samples were additionally tested using nested PCR targeting the *gltA* gene.

**Fig. 2:**
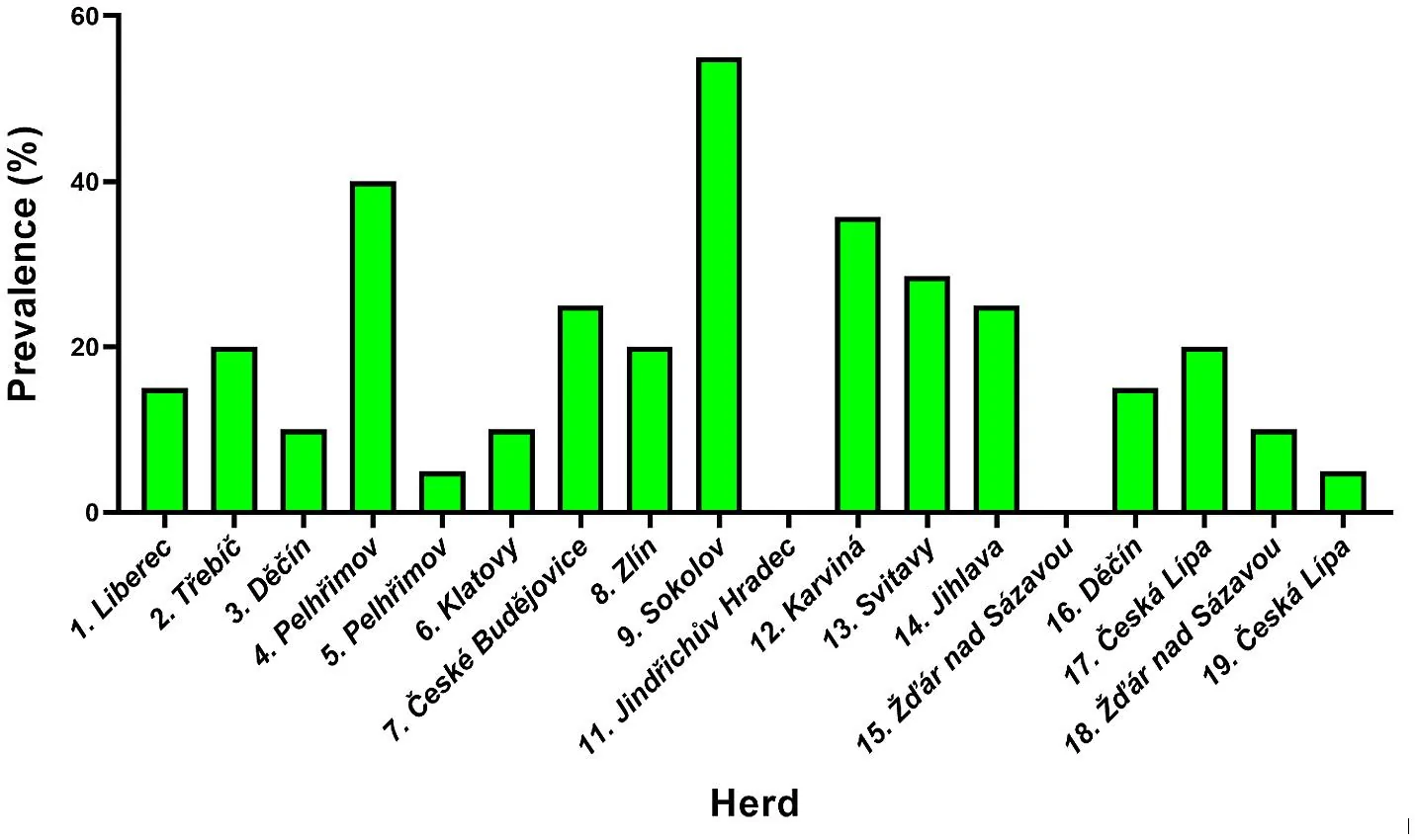
Percentage of positive cattle in individual pasture-raised herds; most herds included 20 animals, whereas herds 12 and 13 included 14 animals each. The overall prevalence across herds was 18%.

Of the 348 pasture-raised cattle samples, 64 were positive, corresponding to an overall prevalence of 18.39%. Of the 18 pasture herds, *B. bovis* was detected in 89%. The highest prevalence was observed in herd 9 (55%), while herds 11 and 15 were negative (see Fig. 2), which may reflect differences in vector presence or specific herd locations. The prevalence of *Bartonella* in pasture-raised cattle (18%) is comparable to reports from other countries, which show a wide range: 28% in Brazil (Gonçalves et al., 2020) and 24% in Israel (Gutiérrez et al., 2014), while in Europe, lower to moderate prevalence values have been reported: 6.8% in Poland (Welc-Falęciak and Grono, 2013), 35% in France (Bermond et al., 2002), and 24% in Italy (Martini et al., 2008).

Analysis of 22 randomly selected gltA nested PCR sequences confirmed that all but one case represented an identical *B. bovis* genotype (GenBank Accession Number: PX830971), fully matching isolates from Brazilian cattle (MN615930, MN615927, MN615924; (Gonçalves et al., 2020)), and strains from Guatemala (KF199897) and France (KF199895; (Bai et al., 2013)). The remaining sequence (PX830972) differed by one nucleotide. The external comparison cohort included 48 blood samples from cattle in southeastern Romania. Eleven of these were positive (12.5% prevalence). Nine of these isolates were 100% identical (PX830973) to the majority Czech isolate, while one sample (PX830974) matched 100% with an isolate from Brazilian cattle (MN615935; (Gonçalves et al., 2020)) and a strain from Guatemala (KF199896; (Bai et al., 2013)). One sample contained a mixed infection of these two *B. bovis* genotypes.

The most important finding of the study, however, was the analysis of blood samples from twenty indoor herds. All tested cows were negative. The difference in prevalence is statistically highly significant (Fisher's exact test, *p* < 0.0001) and provides hints about the ecology of the vector.

## 4 Discussion

The dominance of a single *B. bovis* genotype in Czech and Romanian herds stands in sharp contrast to the high genetic variability observed in *B. schoenbuchensis* from red deer (*Cervus elaphus*) in the same region (Hammerbauerová et al., 2025). Only one study has investigated the genetic diversity of *B. bovis* using multi-locus sequence typing (MLST), analyzing 28 strains from different countries and identifying 22 sequence types (Bai et al., 2013). This study showed that cattle isolates belong to lineages I and II, while water buffalo isolates fall into lineage III. Although the different sequence types are very closely related (0.1–1.8% divergence), they have never been found across regions, suggesting regional variability potentially linked to cattle breed. The majority isolate in both Czech and Romanian cattle belongs to lineage I and is 100% identical to two sequence types from France and Guatemala (these two types are identical for *gltA*, tested in our study, but show minor differences in other MLST genes), indicating a probable clonal population structure. A distinct Romanian strain, represented by a single sample, belongs to lineage II (sequence type from Brazil and Guatemala). Compared to the diversity of *B. schoenbuchensis*, the clonality of *B. bovis* in cattle is surprising. The only significantly different genotype, or possibly subspecies, of *B. bovis* was detected in moose and deer (Duodu et al., 2013; Hammerbauerová et al., 2025). The homogeneity observed in cattle may reflect more stable association with one host, as opposed to several hosts in the case of *B. schoenbuchensis*.

The relatively high prevalence of *B. bovis* in pasture-raised cattle, compared with the complete absence of *Bartonella* in indoor herds, supports transmission via exophagic vectors. A possible effect of insecticide or ectoparasiticide treatments cannot be completely excluded, as detailed farm-level data on their use were not available for all herds. Selective insecticide treatment, as well as the specific microclimate of the pasture, may contribute to the disparity in pasture herd prevalence, such as the absence of Bartonella in herds 11 and 15. However, such treatments are applied selectively in both indoor dairy and pasture-based cattle systems in Czechia, depending on specific farm issues, and are not expected to systematically account for the complete absence of *B. bovis* in indoor herds. Therefore, differences in exposure to exophagic pasture-associated vectors remain the most plausible explanation. Pasture-raised cattle are exposed to a broader spectrum of blood-feeding arthropods, including exophagic (outdoor-biting) vectors, such as horseflies (Tabanidae), black flies (Simuliidae), certain louse flies (Hippoboscidae), horn flies (*Haematobia irritans*), as well as ticks (Ixodidae) present on pastures. In contrast, indoor cattle are protected from these exophagic vectors, although they are still exposed to other blood-feeding ectoparasites which are not exclusively exophagic, such as lice (*Haematopinus*, *Linognathus*), biting midges (*Culicoides*), mosquitoes (mainly *Anopheles*, *Aedes*, *Culiseta*), mites (Dermanyssidae), and stable flies (*Stomoxys calcitrans*).

Louse flies of the genus *Hippobosca*, in which the related *B. schoenbuchensis* subsp. *chomelii* has been detected (Halos et al., 2004), are not present in the region. Ticks, particularly *Rhipicephalus microplus*, have been shown to carry *B. bovis* in Taiwan (Tsai et al., 2011); however, this species does not occur in Central Europe, and *Bartonella* has not been detected in the common tick *Ixodes ricinus* in the region (unpublished data). A study testing black flies (Simuliidae) in Czechia did not detect any *B. bovis* DNA, so their involvement in transmission is also unlikely (Šikutová et al., 2026). Potential competent vectors of *B. bovis* in Central Europe therefore include horn flies or horse flies. Horn flies and horse flies were tested in only one study, in which one of 45 horn fly pools tested positive for *B. bovis* DNA, and all eleven horse fly individuals were negative (Chung et al., 2004). As per the authors' suggestion, however, this may correlate with low *Bartonella* prevalence in cattle in the tested area. To determine the vector for *B. bovis*, molecular testing of blood-sucking arthropods in areas with high prevalence should be carried out. While laboratory experiments on cows would be complicated, competence testing through xenodiagnosis could confirm intake of bacteria by suspected vectors.

## Statement on AI use

No generative AI was used in the preparation of this manuscript.

## CRediT authorship contribution statement

**Iva Hammerbauerová:** Writing – review & editing, Writing – original draft, Methodology, Investigation, Formal analysis. **Kristýna Dulavová:** Methodology, Investigation, Formal analysis, Data curation. **Petr Václavek:** Resources. **Jan Votýpka:** Writing – review & editing, Writing – original draft, Supervision, Resources, Project administration, Conceptualization.

## Ethical statement

Ethical review and approval were not required for this study, as all animal samples were collected by professional veterinarians during routine animal health monitoring.

## Funding

This research was funded by the Ministry of Health, Czech Republic (grant number NU2305–00511) and the Charles University Grant Agency, Prague, Czech Republic (grant number 284423).

## Declaration of competing interest

The authors declare that they have no known competing financial interests or personal relationships that could have appeared to influence the work reported in this paper.
